# New Insights into Molecular Mechanisms and Radiomics in Non-Contrast CT for Aortic Dissection: A Case Report and Literature Review

**DOI:** 10.3390/life16010014

**Published:** 2025-12-22

**Authors:** Jian-Cheng Tian, Jia-Hao Zhou, Jui-Yuan Chung, Po-Chen Lin, Giou-Teng Yiang, Ya-Chih Yang, Meng-Yu Wu

**Affiliations:** 1 Department of Emergency Medicine, Taipei Tzu Chi Hospital, Buddhist Tzu Chi Medical Foundation, New Taipei 231, Taiwan; diegowu06@gmail.com (J.-C.T.); taipeitzuchier@gmail.com (P.-C.L.); gtyiang@gmail.com (G.-T.Y.); 2 School of Medicine, Tzu Chi University, Hualien 970, Taiwan; charles198217@gmail.com; 3 Department of Medical Imaging, Taipei Tzu Chi Hospital, Buddhist Tzu Chi Medical Foundation, New Taipei 231, Taiwan; 4 Department of Emergency Medicine, Cathay General Hospital, Taipei 106, Taiwan; bybarian@gmail.com; 5 School of Medicine, Fu Jen Catholic University, New Taipei 242, Taiwan; 6 School of Medicine, National Tsing Hua University, Hsinchu 300, Taiwan

**Keywords:** aortic dissection, non-contrast computed tomography, computed tomography angiography, intima flap

## Abstract

**Background:** Computed tomography (CT) angiography is widely regarded as the gold standard for diagnosing acute aortic dissection. However, in patients with contraindications to iodinated contrast media, such as those with renal insufficiency or hemodynamic instability, non-contrast CT may offer a viable alternative for initial evaluation. Understanding the molecular mechanisms underlying aortic dissection, including extracellular matrix degradation, smooth muscle cell apoptosis, and inflammatory pathways, is crucial for developing novel diagnostic and therapeutic approaches. This report describes a single case of acute Stanford type A aortic dissection initially detected on non-contrast CT. **Case Presentation:** We describe a 74-year-old man who presented to the emergency department with fever and suspected infection, but without chest pain. An incidental finding on non-contrast CT revealed ascending aortic dilatation, pericardial effusion, and a suspected intimal flap. Subsequent CT angiography confirmed a Stanford type A aortic dissection. **Conclusions:** This case highlights the potential value of non-contrast CT in the early detection of aortic dissection, particularly when CT angiography cannot be performed. Recent advances in artificial intelligence (AI) and radiomic analysis have shown promise in augmenting the diagnostic capabilities of non-contrast CT by identifying subtle imaging features that may correlate with underlying molecular pathology and elude human observers. Emerging evidence suggests that radiomic features may reflect molecular alterations in the aortic wall, including metalloproteinase activity, collagen degradation, and inflammatory cell infiltration. Incorporating AI-assisted interpretation alongside insights into molecular mechanisms could facilitate earlier diagnosis, improve risk stratification, and guide personalized treatment strategies in critically ill patients. Although non-contrast CT has limited sensitivity for aortic dissection, it may still reveal crucial findings in selected cases and should be considered when contrast-enhanced imaging is not feasible. Ongoing progress in AI, radiomics, and molecular biomarker research may further expand the clinical applications of non-contrast CT in emergency cardiovascular care and bridge the gap between imaging phenotypes and molecular endotypes. These findings are hypothesis-generating and require validation in larger cohorts before clinical generalization.

## 1. Introduction

Aortic dissection is a rare but life-threatening cardiovascular emergency with an incidence of 2.6–3.5 per 100,000 years, predominantly affecting males aged 60–80 [1,2]. Most cases involve the ascending aorta (Stanford type A) and are associated with high risks of rupture, tamponade, and death, with mortality rising 1–2% per hour if untreated [1,3]. Although imaging techniques have improved, diagnosis remains challenging due to variable presentations; while severe “tearing” chest or back pain is classic, atypical symptoms often lead to delays.

Computed tomography angiography (CTA) is the gold standard for diagnosing acute aortic dissection, with sensitivity and specificity approaching 100% [4]. However, its use may be limited in patients with hemodynamic instability or contraindications to iodinated contrast agents, such as renal insufficiency or allergy. Although transthoracic echocardiography (TTE) and transesophageal echocardiography (TEE) can compensate for the limited feasibility of CTA in certain clinical scenarios, their diagnostic accuracy is highly dependent on operator experience and the quality of the acoustic window. In recent years, advances in computed tomography (CT) hardware and software have enhanced the utility of non-contrast CT in the evaluation of acute aortic dissection (AD), particularly with the integration of artificial intelligence (AI)-based prediction models. Several studies have demonstrated the growing role of non-contrast CT in detecting AD, especially in patients with atypical presentations or concurrent shock etiologies such as sepsis, allowing for simultaneous investigation of shock origin and early identification of AD [4,5,6,7,8,9,10,11,12,13,14,15,16,17,18,19,20,21]. In many patient populations, non-contrast CT can rapidly assist in the diagnosis of AD. Amelie Spangenberg et al. [4] reported a case of a young immunocompromised male patient presenting primarily with acute abdominal pain and renal impairment, highlighting the potential clinical value of non-contrast computed tomography (CT) in detecting aortic dissection. The study further pointed out that on non-contrast CT images, displaced calcified intimal flap, intraluminal high-density sign, intramural hematoma of the aortic wall, and aneurysmal dilatation of the aorta are all common and important imaging features suggestive of aortic dissection. Shinsuke Takeuchi et al. reported 3 cases of out-of-hospital cardiopulmonary arrest patients, all diagnosed with type A acute aortic dissection by non-contrast CT. Non-contrast CT possesses the advantages of being rapid, convenient, and non-invasive, and holds important clinical and research value in investigating the causes of sudden death [14]. However, the specific radiological features of AD identifiable on non-contrast CT have not yet been extensively characterized in the literature.

Here, we report a case of Stanford type A aortic dissection incidentally identified on non-contrast CT in a 74-year-old male who presented with fever and a clinical suspicion of infection. Despite the absence of typical chest pain, non-contrast CT revealed aortic root dilatation, pericardial effusion, and a suspected intimal flap. This case highlights the diagnostic challenges associated with atypical presentations of aortic dissection and underscores the potential role of non-contrast CT in its early detection, particularly in patients for whom contrast administration is contraindicated.

## 2. Case Presentation

A 74-year-old man presented with intermittent fever for 3 days, accompanied by cough, throat pain, chills, headache, shortness of breath, diarrhea, and oliguria. He denied chest, back, or abdominal pain, as well as nausea or vomiting. Past medical history included thoracic aortic aneurysm (5.3 cm), hypertension, and transient ischemic attack on aspirin. Thoracic aortic aneurysm was under regular follow-up for five years under medication controlled. On physical examination, his temperature was 36.5 °C, blood pressure 111/82 mmHg, heart rate 124 bpm, respiratory rate 18/min, and oxygen saturation 94% on room air. The Glasgow Coma Score (GCS) was awareness and alert (E_4_V_5_M_6_). The breath sound was bilateral coarse and abdomen was soft without local tenderness. An irregular heartbeat without murmur and cold extremities were noted.

Initial laboratory data in Table 1 revealed a white blood cell count of 9.27 × 10^9^/L with a markedly elevated neutrophil ratio (82.9%) and decreased lymphocyte percentage (11.5%), indicating acute systemic inflammation. C-reactive protein (CRP) was significantly elevated at 13.9 mg/dL, and lactate was 5.8 mmol/L, consistent with systemic inflammatory response and tissue hypoperfusion. Renal function was impaired, with serum creatinine elevated to 1.85 mg/dL. Cardiac biomarkers were notable for high-sensitive troponin I of 154.6 ng/L and NT-proBNP of 8488 pg/mL, indicating myocardial injury and acute heart failure. COVID-19 and influenza tests were both negative.

**Figure 1:**
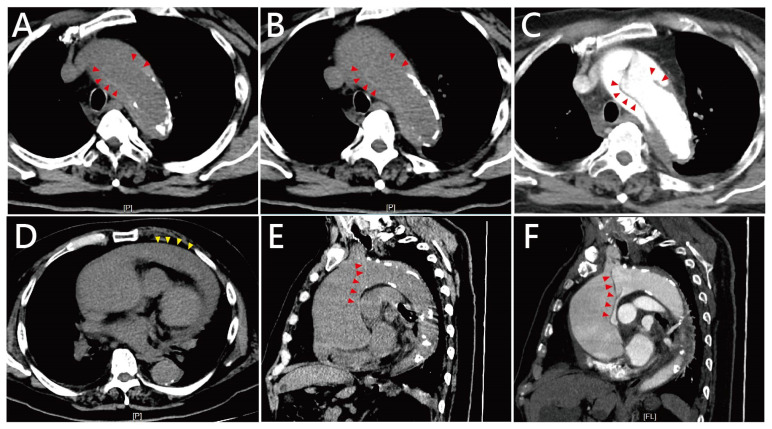
Computed tomography (CT) findings of Stanford type A aortic dissection in this patient. (**A**,**B**) Axial non-contrast CT images at different levels of the ascending aorta demonstrate marked aortic dilatation and an obvious intimal flap (red arrowheads) with associated high-attenuation density, consistent with acute dissection. Hounsfield unit (HU) values of both the suspected inner membrane (HU up to 70–80). (**C**) The presence of the intimal flap is further confirmed by contrast-enhanced CT angiography (red arrowheads). (**D**) An axial CT slice reveals massive fluid accumulation in the pericardial space (yellow arrowheads), compatible with hemopericardium. (**E**,**F**) Sagittal reconstructions from both non-contrast CT (**E**) and contrast-enhanced CT angiography (**F**) show the extent of ascending aortic dilation and the eccentric intimal flap (red arrowheads) coursing along the aortic wall.

**Figure 2:**
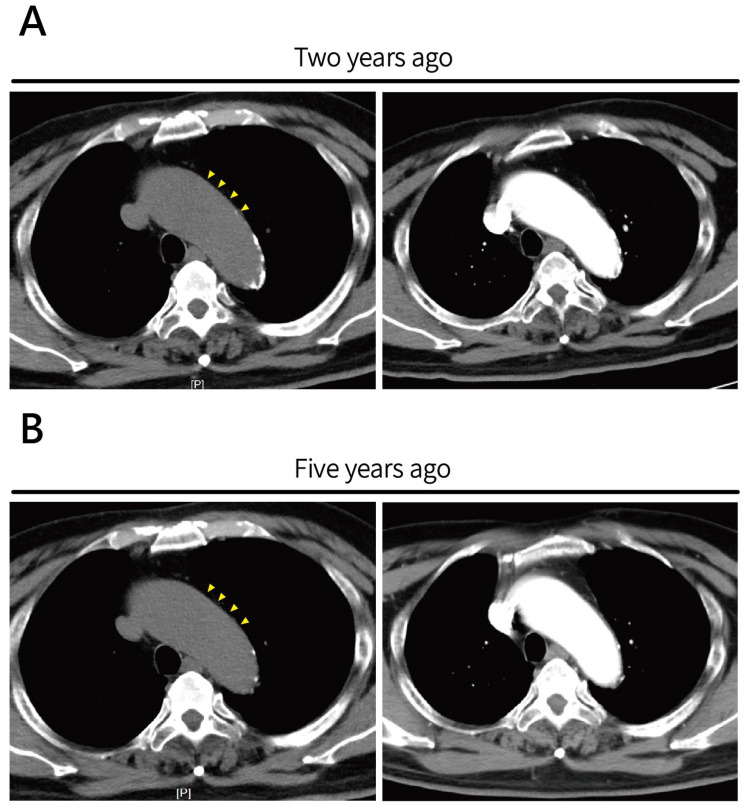
Prior surveillance axial non-contrast CT images and CTA images for ascending aortic aneurysm. (**A**) Image obtained two years prior and (**B**) five years prior demonstrate progressive ascending aortic dilatation over time. No obvious high-attenuation calcification intimal flap or signs of acute aortic pathology were noted at either time point. Retrospective review revealed mild dilatation at the aortic root (yellow arrowheads).

A chest X-ray revealed cardiomegaly and mediastinal widening—findings that were similar compared to a chest radiograph taken five years prior. Due to ongoing hemodynamic instability and evidence of organ dysfunction, a non-contrast CT scan of the chest and abdomen was initially performed to identify a potential source of infection, but it incidentally revealed dilation of the ascending aorta and pericardial effusion in Figure 1. A clearly defined intimal flap with associated high-attenuation density, consistent with classic findings of Stanford type A aortic dissection, was noted. The presence of the intimal flap was further confirmed on contrast-enhanced CT angiography and also confirmed not a calcification flap. Additionally, massive fluid accumulation in the pericardial space, compatible with hemopericardium. Sagittal reconstructions from both non-contrast and contrast-enhanced scans highlighted the extent of ascending aortic dilation and the eccentric intimal flap tracking along the aortic wall. Prior surveillance non-contrast CT images obtained two and five years earlier showed progressive aortic dilatation over time without evidence of high-attenuation intimal flap or acute aortic pathology, supporting the acute onset of the current dissection (Figure 2). There was no additional CT imaging was performed within six months prior to the index event, as the patient was clinically stable and under routine outpatient follow-up without imaging indication.

After the completion of CTA, a consultation with cardiovascular surgery was arranged. However, the patient subsequently experienced a sudden loss of consciousness followed by pulseless electrical activity (PEA). Advanced Cardiac Life Support (ACLS) was immediately initiated. Pericardiocentesis was performed and yielded approximately 10 mL of blood, but return of spontaneous circulation (ROSC) was not achieved. The patient was pronounced dead approximately two hours after arrival at the emergency department.

## 3. Discussion

**Figure 3:**
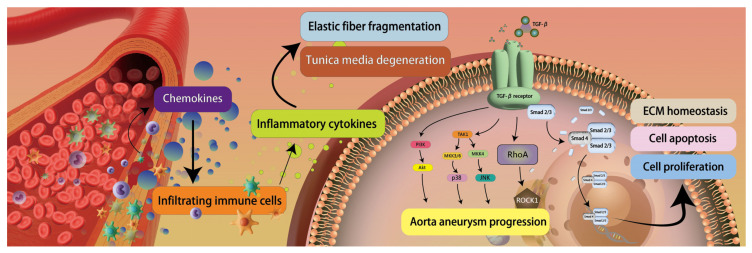
Aortic dissection pathogenesis. Injured vascular smooth muscle cells secrete chemokines, promoting inflammatory cell infiltration into the aortic wall, including T lymphocytes and monocytes. Activated dendritic cells release chemotactic mediators that recruit macrophages and CD4^+^ T cells. These immune cells secrete proinflammatory cytokines, resulting in elastic fiber fragmentation and medial degeneration. Imbalance between proteases and their inhibitors enhances proteolytic activity, impairing extracellular matrix remodeling. The TGF-β signaling pathway regulates cell proliferation, apoptosis, and matrix homeostasis through the Smad2/3-Smad4 complex, while its noncanonical pathways (ROCK and MAPK) also participate in aortic aneurysm progression and dissection pathogenesis, ultimately leading to structural deterioration of the aortic wall. (Figure 3 is an original schematic based on published literature).

Aortic dissection represents an acute and highly lethal cardiovascular emergency characterized by a tear in the intimal layer of the aorta, which allows blood to penetrate into the medial layer of the aortic wall, thereby creating a false lumen and separating the vessel wall into inner and outer layers. This pathological process may propagate along the longitudinal axis of the vessel, affecting multiple aortic segments and consequently compromising perfusion of critical branch vessels. It may ultimately lead to fatal complications, including aortic rupture, cardiac tamponade, acute aortic valve insufficiency, or vital organ ischemia. The pathophysiological mechanisms underlying aortic dissection are complex and multifactorial. Hypertension constitutes the most significant risk factor, as sustained hemodynamic pressure subjects the aortic wall to excessive mechanical stress, thereby precipitating intimal tears. Recent investigations have demonstrated that medial degeneration is closely associated with oxidative stress and related pathological processes. Injured or stressed vascular smooth muscle cells (SMCs) secrete chemokines, notably monocyte chemoattractant protein-1 (MCP-1), which facilitate the recruitment of inflammatory cells into the aortic wall [22]. Consistent with this observation, elevated levels of CD3^+^, CD4^+^, CD8^+^, and CD45^+^ T lymphocytes, as well as CD68^+^ monocytes, have been documented in aortic tissue specimens from patients with aortic dissection [22,23]. Furthermore, activated dendritic cells residing at the adventitia–media interface release chemotactic mediators that augment the recruitment of macrophages and CD4^+^ T cells. These infiltrating immune cells subsequently undergo clonal expansion and secrete proinflammatory cytokines—including tumor necrosis factor-α (TNF-α), interferon-γ (IFN-γ), interleukin (IL)-1, IL-2, IL-6, and IL-8—which modulate macrophage differentiation and effector functions [24,25]. Sustained activation of these inflammatory cascades culminates in elastic fiber fragmentation and progressive medial degeneration [26]. Hypoxia and augmented oxidative stress further exacerbate medial degradation. Investigations have revealed elevated concentrations of malondialdehyde, an oxidative stress biomarker, alongside diminished levels of extracellular superoxide dismutase, a principal antioxidant enzyme, in TAD patients [27]. The intensification of inflammation and oxidative stress promotes SMC apoptosis, while dysregulation between proteases and their endogenous inhibitors enhances proteolytic activity and compromises extracellular matrix (ECM) remodeling, thereby accelerating SMC depletion and structural deterioration of the aortic wall. The transforming growth factor-β (TGF-β) signaling pathway additionally contributes to ECM remodeling and degradation. Following ligand binding, TGF-β receptors (TGFBRs) undergo activation and, via the canonical pathway, phosphorylate Smad2 and Smad3, which subsequently recruit Smad4 to constitute the Smad2/3–Smad4 transcriptional complex [28]. This complex translocates to the nucleus and induces transcription of TGF-β target genes implicated in cell proliferation, apoptosis, and ECM homeostasis. Elevated TGF-β activity may also derive from noncanonical signaling pathways, including the Rho-associated protein kinase (ROCK) and mitogen-activated protein kinase (MAPK) cascades, both of which have been implicated in aortic aneurysm progression and are likely pertinent to aortic dissection pathogenesis [29,30] (Figure 3). In this case, no serum or tissue molecular testing was performed. The related molecular mechanisms were inferred based on existing literature rather than actual measurements from the individual case. In the future, in addition to using non-contrast CT for rapid diagnosis of AD, further validation could be considered through candidate biomarkers, including MMP-9, IL-6, IL-1β, and indicators related to inflammatory response and extracellular matrix remodeling.

Echocardiography (Echo) is the most commonly used tool for initial assessment because it possesses numerous advantages, including being a non-invasive, readily available, and easy-to-use fundamental diagnostic tool. Routine aortic ultrasound assessment can adopt the “four Ss” approach [31]—Superior intercostal view, Small-scale long-axis view, Subxiphoid view, and Suprasternal view—performed in both the “sniff” and supine positions, to rapidly examine the ascending aorta, descending aorta, abdominal aorta, and aortic arch. In diagnosing acute aortic dissection, transthoracic echocardiography (TTE) can easily detect pericardial effusion, which is a common complication of type A aortic dissection, while the intimal flap within the aorta is an important feature for identifying aortic dissection. Compared to TTE, transesophageal echocardiography (TEE) overcomes the limitation of TTE’s narrow assessment window and can be performed simultaneously during resuscitation of OHCA patients, demonstrating high accuracy in diagnosing acute aortic dissection (with sensitivity of approximately 98%). Operator dependence of echocardiography should also be considered when interpreting its diagnostic performance in suspected aortic dissection. Both TTE and TEE are highly operator-dependent modalities, with diagnostic accuracy influenced by the examiner’s experience, image acquisition technique, patient body habitus, and institutional expertise. In emergent or atypical clinical presentations, suboptimal acoustic windows or limited examination time may further reduce sensitivity, particularly for distal ascending or arch involvement. While TEE generally provides higher spatial resolution and improved visualization of the proximal aorta compared with TTE, its availability and timely application may be constrained in unstable patients or resource-limited settings. In this context, non-contrast CT may serve as a complementary imaging modality by providing rapid, operator-independent anatomic information and by identifying suggestive features that prompt further definitive evaluation with CT angiography. Therefore, echocardiographic findings should be interpreted within the broader clinical context and in conjunction with cross-sectional imaging, rather than as standalone diagnostic tools [31].

Computed Tomography Angiography (CTA) is widely regarded as the gold standard for diagnosing aortic dissection due to its high sensitivity and specificity. However, its application may be limited in certain clinical scenarios. In patients with renal insufficiency or known contrast allergies, the administration of iodinated contrast agents carries the risk of contrast-induced acute kidney injury (CI-AKI), potentially delaying definitive interventions. Although bedside echocardiography is often used as an alternative, it has inherent limitations. It is highly operator-dependent and may not provide the comprehensive anatomical detail required by cardiovascular surgeons, leading to lower diagnostic sensitivity and specificity compared to CTA. Furthermore, CTA requires the placement of a relatively large-bore intravenous (IV) catheter to ensure adequate contrast bolus delivery. In patients with difficult vascular access—such as those in shock—this requirement often results in delayed diagnosis. In critically ill patients with only intraosseous (IO) access and no usable IV line, CTA becomes infeasible. In such circumstances, non-contrast CT offers a time-efficient alternative, eliminating the need for IV placement and contrast administration. Although its sensitivity is limited, non-contrast CT may serve as a valuable initial screening tool in unstable or contrast-contraindicated patients.

Non-contrast 4-dimensional phase-contrast magnetic resonance imaging (4D PC-MRI) is a newly developed technique that can acquire blood flow information of the entire aortic volume over time and visualize the relationships between the false lumen, true lumen, major visceral branches, and primary entry tears connecting the true and false lumens. Chien-Wei Chen et al. [32] reported that 4D PC-MRI, without using radiation or contrast agents, can provide aortic information comparable to contrast-enhanced CTA in patients diagnosed with type B aortic dissection who have not undergone interventional treatment and in patients with residual dissection following open surgical repair of type A aortic dissection. However, endograft materials, particularly stainless steel, may limit the further application of 4D PC-MRI. Furthermore, not all medical facilities provide 24 h MRI services, and the relatively lower spatial resolution results in inferior visualization of small intimal tears compared to CTA [33,34,35,36]. Additionally, in patients with implanted devices such as pacemakers, the devices must be evaluated or deactivated, rendering MRI less clinically practical than bedside ultrasound and CTA [37].

In past reports, common non-contrast CT findings of aortic dissection include displaced calcified intimal flaps, intraluminal high densities, intramural hematomas, and aneurysmal aortic dilatation [38]. The diagnostic criteria based on the presence of intimomedial flap or intramural hematoma, inward shift of the calcified intima, and double sedimentation in the true and false lumen caused by hypostasis [14]. Displaced calcified intimal flaps and crescentic hyperattenuating intramural fluid collections are among the most commonly observed features of aortic dissection on non-contrast CT. In contrast, the visualization of an intimomedial flap often requires a stable and cooperative patient during image acquisition, as motion artifacts can easily obscure this subtle finding. In previous studies investigating the use of non-contrast CT to identify the cause of death in out-of-hospital cardiac arrest (OHCA) patients, the most frequently observed findings included “crescentic hyperattenuating intramural fluid collection,” followed closely by “intimal flap” and “inward displacement of calcified intima” [14]. This may be attributed to the cessation of cardiac motion after death, which renders the intimal flap stationary and thereby more easily visualized on imaging.

The diagnostic accuracy of non-contrast CT scans for subtle findings is highly dependent on patient cooperation, particularly when evaluating the intimal flap of an aortic dissection. In patients experiencing severe pain, it may be difficult to remain still during the scan, leading to motion artifacts that obscure the subtle density differences between the intimal flap and the aortic blood flow. Additionally, image acquisition during diastole—when the vena cava and aorta are filled with blood—may increase the likelihood of visualizing these subtle contrasts. In our case, two CT scans were performed. During the first non-contrast CT scan, the intimal flap was clearly visualized. However, in the second scan, performed immediately prior to CTA, the intimal flap was no longer identifiable on the non-contrast images. This discrepancy was likely due to the patient’s inability to remain still during the second scan because of worsening pain, resulting in motion that diminished the detectable density difference between the flap and the aortic lumen. This observation highlights the importance of image quality when using non-contrast CT to diagnose aortic dissection. It also underscores the potential role of artificial intelligence in overcoming such limitations and enhancing diagnostic robustness in challenging clinical conditions.

Previous studies have suggested that imaging data of different dimensions (e.g., 2D vs. 3D) can provide complementary structural and spatial information, thereby enhancing the accuracy of disease detection and classification. A study by Hata et al. [9] developed a 2D deep learning (DL) model for detecting aortic dissection on non-contrast CT, achieving an AUC of 0.940, with performance comparable to radiologists. The lack of 3D spatial information in the model appears to limit its applicability in clinical practice. Yan Yi et al. [19] addressed these limitations by implementing a 3D model, which achieved superior performance with an AUC of 0.948 on the internal testing set and 0.969 on the external testing set. However, in our case, the reconstructed 3D images also clearly demonstrated the trajectory of the intimal flap on non-contrast CT. Nevertheless, due to suboptimal image quality, the information provided by the reconstructed images was not readily identifiable, and the flap could easily be mistaken for noise and overlooked. Therefore, regardless of whether a 2D or 3D model is used, the critical determinant of diagnostic performance remains the quality of the original 2D images.

With the rapid advancement of AI and medical image processing technologies, there has been growing interest in leveraging AI algorithms, radiomics, and image synthesis techniques to enhance the diagnostic performance of non-contrast CT in detecting aortic dissection, particularly in patients for whom contrast administration is contraindicated. From the AI perspective, various deep learning architectures have been applied to non-contrast-CT-based aortic dissection detection with promising results. These include object detection and classification models such as YOLO, Xception, U-Net, DenseNet, and ResNet, as well as architectures incorporating attention mechanisms like the Convolutional Block Attention Module (CBAM) [9,39,40,41,42]. A previous study reported that a CBAM-enhanced YOLOv5 model achieved an area under the curve (AUC) of 0.938, with an accuracy of 91.5%, sensitivity of 90.0%, and specificity of 92.9%. While the baseline model demonstrated sensitivity comparable to that of experienced radiologists, the proposed CBAM-based algorithm showed superior overall detection performance and demonstrated strong potential for clinical deployment [7].

Radiomics has also emerged as a valuable complementary tool, enabling the extraction of high-dimensional features—such as texture, shape, and intensity histograms—from non-contrast CT images. By applying least absolute shrinkage and selection operator (LASSO) regression for feature selection, radiomic signatures with high predictive value for aortic dissection have been developed. Several studies have reported models achieving AUCs greater than 0.95 and high negative predictive values, underscoring their utility in ruling out aortic dissection in challenging cases [21]. In the field of image synthesis, Generative Adversarial Networks (GANs) offer an innovative approach by generating contrast-like images from non-contrast CT scans, enabling visualization of vascular structures without the need for iodinated contrast agents [43]. These methods can effectively bridge the gap in diagnostic imaging for patients who cannot undergo CTA. Collectively, these advances demonstrate that integrating AI with non-contrast CT imaging holds great promise as a front-line screening and diagnostic support tool for aortic dissection, especially in high-risk or contrast-ineligible populations. As these technologies continue to evolve, they may further strengthen the speed, accuracy, and accessibility of emergency imaging and clinical decision-making.

This study has several limitations that should be acknowledged. First, this report describes a single clinical case, which inherently limits the generalizability of the observations and precludes any causal inference. Second, no patient-specific molecular testing, radiomic feature extraction, or artificial intelligence-based analysis was performed; therefore, the discussion of molecular mechanisms and AI/radiomics is based solely on previously published literature and should be regarded as hypothesis-generating rather than confirmatory. Third, the imaging findings were interpreted retrospectively, and subtle non-contrast CT features may be subject to observer dependency and selection bias. Fourth, the absence of systematic longitudinal imaging and standardized follow-up data limits the ability to accurately assess disease progression over time. Finally, although non-contrast CT demonstrated suggestive findings in this case, CT angiography remains the diagnostic gold standard, and the potential clinical utility of non-contrast CT as an adjunctive tool requires validation in larger, prospective cohorts.

## 4. Conclusions

This case highlights the diagnostic potential and limitations of non-contrast CT in detecting Stanford type A aortic dissection, particularly in patients for whom contrast administration is contraindicated. The initial scan revealed a clear intimal flap on non-contrast CT, emphasizing its value as an early screening tool. However, the second non-contrast CT failed to demonstrate the flap, likely due to patient motion and compromised image quality. This underscores the critical role of image acquisition conditions in non-contrast CT interpretation and suggests that both 2D and 3D deep learning models are highly dependent on the underlying image quality. As artificial intelligence continues to evolve, its integration with non-contrast CT may enhance diagnostic robustness, especially in emergency settings involving high-risk patients with limited imaging options.

## Figures and Tables

**Table 1:** The basic laboratory analysis in this patient.

Variables	Patient Data		Reference Value
White cell count	9.27	--	3.5–11 (×10^9^/L)
Neutrophil	82.9%	↑	40–75%
Lymphocyte	11.5	↓	40–45%
Monocyte	5.2	--	2–10%
Eosinophil	0.2	↓	1–6%
Hemoglobin	13.2	--	12–16 g/dL
Platelet counts	126	↓	150,000–400,000/uL
Creatinine	1.85	--	0.55–1.02 mg/dL
Sodium	136	↓	136–145 mmole/L
Potassium	3.6	--	3.5–5.1 mmole/L
Glucose	227	↑	70–100 mg/dL
Alanine aminotransferase	21	--	16–63 U/L
Total bilirubin level	1.64	↑	0.3–1.0 mg/dL
Lipase	15	--	11–82 U/L
High-sensitive Troponin I	154.6	↑	0–19 ng/L
Creatine Kinase	359	↑	30–233 U/L
Creatine Kinase MB form	1.2	--	0.6–6.3 ng/mL
NT-proBNP	8488	↑	<450 pg/mL
Lactate	5.8	--	0.4–2.0 mmol/L
C-Reactive Protein	13.9	↑	0–0.33 mg/dL
COVID test	negative	--	negative
Influenza test	negative	--	negative
Vein blood gas test			
pH	7.356		7.31–7.41
pCO_2_	41.3	--	41–51 mmHg
pO_2_	22.3	↓	30–40 mmHg
HCO_3_^−^	22.6	--	22–26 mmol/L
ctCO_2_	23.8	--	23–27 mmol/L
Base Excess	−4.5	↓	−3.3–2.3 mmol/L
Bfecf	−2.9	--	mmol/L
SBC	22	--	22–26 mmol/L
O_2_ saturation	34.1%	↓	

NT-proBNP: N-terminal pro-B-type natriuretic peptide; --: no upper or below low limit of reference range.

## Data Availability

The data presented in this study are available on request from the corresponding author due to privacy or ethical restrictions.
